# WHO World Report on Hearing: Implications for the United States and the WHO Decade of Healthy Aging

**DOI:** 10.1093/geroni/igaa057.2931

**Published:** 2020-12-16

**Authors:** Carrie Nieman

**Affiliations:** Johns Hopkins University School of Medicine, Baltimore, Maryland, United States

## Abstract

The past 5 years have seen incredible advances in approaching hearing loss as a major public health issue. National efforts include the 2015 President’ Council of Advisors on Science and Technology and the National Academies of Science, Engineering, & Medicine’s 2016 Commission on Hearing Health Care for Adults, which led to the 2017 OTC hearing aid legislation and the expected debut of OTC hearing aids in 2020-2021. The World Report on Hearing amplifies these efforts. This presentation will cover the role of the Report in the context of the rapidly evolving hearing care landscape in the US and how the Report’s call for affordable, accessible hearing care fit within current national efforts focused on older adults. Finally, the WHO recognized 2020-2030 as the Decade of Healthy Aging. We will discuss how the World Report on Hearing integrates with broader efforts to support healthy aging locally and globally.

